# Multi-state outcome analysis of treatment interventions after failure of non-surgical root canal treatment: a 13-year retrospective study

**DOI:** 10.1590/1678-7757-2020-1079

**Published:** 2021-09-03

**Authors:** Pradeep BHAGAVATULA, Alex MOORE, Lisa REIN, Aniko SZABO, Mohamed IBRAHIM

**Affiliations:** 1 Marquette University School of Dentistry Program in Public Health MilwaukeeWisconsin United States Marquette University School of Dentistry, Program in Public Health, Milwaukee, Wisconsin.; 2 Illinois United States Endodontist in private practice in Illinois.; 3 Institute for Health & Equity Division of Biostatistics Medical College of Wisconsin MilwaukeeWisconsin United States Institute for Health & Equity, Division of Biostatistics, Medical College of Wisconsin, Milwaukee, Wisconsin.; 4 Marquette University School of Dentistry Milwaukee Program in Endodontics Wisconsin United States Marquette University School of Dentistry Milwaukee, Program in Endodontics, Wisconsin; Mansoura University, Program in Endodontics, Mansoura, Egypt.; Mansoura University Program in Endodontics Mansoura Egypt

**Keywords:** Root canal therapy, Survival rate, Tooth apex, Treatment outcome

## Abstract

**Objective:**

To examine the factors affecting the transitions through treatment interventions after failure of non-surgical root canal treatment (NS-RCT).

**Methodology:**

Insurance enrollment and claim information for enrollees of Delta Dental of Wisconsin (DDWI), USA were analyzed for 438,487 initial NS-RCT procedures to determine the effect of initial provider type and other covariates on additional treatments (no additional treatment, nonsurgical retreatment, surgical retreatment and extraction). A multi-state model was created using the “mstate” R package. Transitions between the four states identified by Code on Dental Procedures and Nomenclature were analyzed. Cox proportional Hazards regression stratified by transition type was used to estimate the effect of provider type on the risk of each transition, adjusting for covariates.

**Results:**

The overall survival rates for all teeth that were treated by NS-RCT was 82.8% [95% CI 82.57%, 83.11%] at 10 years. Approximately, 7% of cases changed from the first state of initial NS-RCT during the 13-year study period with ultimately 0.9%, 0.4% and 5% of cases receiving non-surgical retreatment, surgical retreatment or extraction, respectively. Teeth are more likely to be retreated non-surgically than surgically, and to be extracted than retreated. In general, the probability of a tooth having non-surgical retreatment was higher if the initial provider was not an endodontist (Hazard Ratio (HR)=3.2). Molars were more likely to be non-surgically retreated (HR=2.0) or extracted (HR=2.8) when compared to anterior teeth. The probability of non-surgical retreatment (HR=0.93) or extraction (HR=0.50) was lower when a crown was placed within 90 days after NS-RCT.

**Conclusion:**

Most teeth remained in the same state after treatment with no additional treatment transitions. When a transition occurred, it was more likely to be an extraction. Type of provider, age, location of the tooth, gender, and time to placement of final restoration significantly influence treatment transitions.

## Introduction

When dental caries or trauma lead to pulp and periapical pathosis, non-surgical root canal treatment (NS-RCT) is often the most common and conservative treatment option available to save the natural tooth. The success rates of endodontic therapy have been reported ranging from 81% to 97%.^1-3^ Previous studies on prognosis of endodontic treatment report that the type of provider, age of the patient, type of the restoration, time gap between completion of root canal treatment and final restoration as factors affecting survival of endodontically treated teeth.^4,5^

NS-RCTs are largely successful; however, a small portion of treatments fail.^6^ Non-healing apical periodontitis after endodontic treatment is an indication of failed treatment. Some of the etiological factors for endodontic failure include intraradicular and/or extraradicular infection, reactions to foreign bodies, and presence of true cysts.^7^ Persistent bacterial infection is the main cause of endodontic failure. Inadequate aseptic control, missed canals, inadequate chemomechanical disinfection, leaking restorations, and extruded debris infected with microorganisms have all been described as causes for persistence of bacterial infection.^7-9^ The human body and the infection interact constantly. The goal of an NS-RCT is to aid the human body in preventing and/or stemming the infection from the root canal system and help in the repair, healing and maintenance of the periapical region of the tooth.

When NS-RCTs fail, secondary endodontic treatments such as non-surgical retreatment or surgical retreatment can be performed to save the tooth, or the affected tooth may be extracted. Previous studies have used these procedures as markers for failure of endodontic treatments. These procedures were described as untoward events and any subsequent treatments were disregarded.^3-5^ Studies have also examined the frequencies of treatments after failure of primary endodontic treatment.^10^However, an endodontically treated tooth might have no additional treatments (successful) or transition to additional treatment states such as non-surgical retreatment, surgical retreatment or extraction. The additional treatments indicate a probable failure of the preceding treatment. These transitions are a common occurrence and can be dictated by the clinical case presentation and patient or provider preferences. No studies have examined the transitions that the teeth take through these states and if the type of provider (endodontist *vs* non-endodontist-general dentists and providers from all other dental specialties) and other covariates such as location of tooth, age of the patient or time to final restoration affect these transitions.

In this study, we used insurance claims and enrollment information from a 13-year period to examine the factors affecting the transition states. Multi-state models (MSM) are generally used to model the outcomes in studies where participants may transition to any or all finite set of events, generally randomly, from one state to the next.^11^The models can provide predictions for multiple outcomes simultaneously. The MSMs also allow us to examine the effect of covariates on the transitions, to estimate the progression and survival rates in transition stages, and even the overall prognosis of the tooth.

Knowing the information about treatment transitions can greatly help in clinical decision making and developing a proper strategy by non-endodontists to refer these cases to specialists for better prognosis. Selection of alternate treatment options should be done based on the best available evidence. The endodontic literature on studies at a high level of evidence regarding decision making on treatment options after failure of NS-RCT is scarce, and the consensus among dental professionals is insufficient when making decisions related to what is next after persistence of periapical lesions and/or symptoms after NS-RCT completed.^12,13^ This study aimed to examine the factors affecting the transitions through treatment interventions after failure of non-surgical root canal treatment (NS-RCT).

## Methodology

The subjects in this study were enrollees of Delta Dental of Wisconsin (DDWI) USA that underwent a non-surgical root canal treatment (NS-RCT) between January 1, 2000 and December 31, 2013. This is the same enrollment and claims database used in our previous study.^4,5^ The dataset contained demographic information of the enrollees, start and end dates of dental insurance coverage, as well as all dental claims with date of service and procedures performed. DDWI is the largest private dental insurance and benefits program with more than 1.25 million enrollees. DDWI has the largest provider network in the state of Wisconsin and 90 percent of Wisconsin’s dentists are registered providers of DDWI’s network and it provides large and small group plans through employers, as well as individual dental plans. It uses network discounted fee schedules for reimbursement of dentists.

In total, 491,915 NS-RCTs were identified on maxillary and mandibular teeth using insurance billing codes [Code on Dental Procedures and Nomenclature (CDT)]. The American Dental Association (ADA) created and regularly updates the CDT codes to enable uniform reporting of dental treatment. The codes are widely used in the United States for insurance billing and reimbursement purposes.

The database was searched for D3310, D3320 and D3330 which represent NS-RCTs of anterior, pre-molar and molar teeth, respectively. We used ninety days after the initial NS-RCT therapy as a landmark to assess the presence or absence of a post/core and or crown. After applying the inclusion and exclusion criteria (only NS-RCTs on permanent teeth, a 90-day continuous insurance coverage after NS-RCT and no evidence of failure in the first 90 days), 438,487 teeth were included in the study. Teeth that did not have at least 90 days of continuous insurance coverage (34,616) and teeth that failed within the 90 days of NS-RCT (3,376) were excluded.

We followed the teeth for evidence of any additional treatment interventions after the initial NS-RCT. Each additional treatment-nonsurgical retreatment (D3346, D3347 and D3348 for anterior, pre-molar and molar teeth, respectively), surgical retreatment (apicoectomy- D3410, D3421 and D3425 for anterior, pre-molar and molar teeth, respectively) and extraction (D7140) was considered a transition state. An NS-RCT treated tooth could potentially have no additional treatment (successful), be retreated, or be extracted. When teeth were retreated, non-surgically or surgically, we continued to follow them for further interventions. The cases were followed for the duration of continuous insurance coverage and considered successful until the CDT codes for one of the transition states. Follow-up was stopped at extraction and censored at the end of continuous insurance coverage or the end of the data coverage on 12/31/2014.The categorization of providers into endodontists and non-endodontists was done using the same criteria as Burry, et al.^4^ (2016).

**Figure 1:**
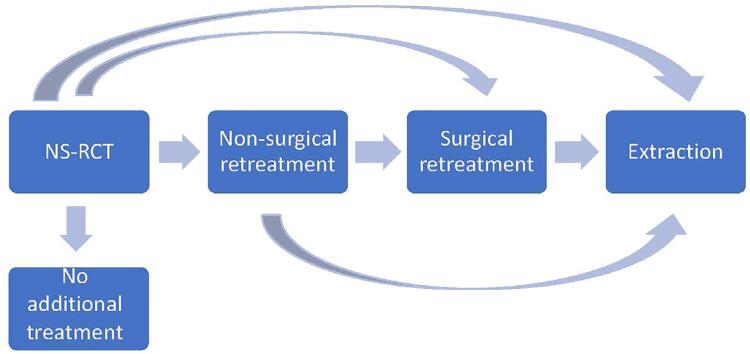
Multi-state model for transitions after NS-RCT completion

We created a multi-state model (MSM) using the “mstate” R package. The model was setup to allow four possible transitions to a higher level of re-intervention (Figure 1). Multi-state modelling is a technique based on survival-analysis and allows non-parametric estimation of the process of transitions between states over time in the presence of censoring. The cumulative hazard and transition probabilities from the model were plotted for all NS-RCT procedures and separately by initial provider type. Formal comparison at selected time-points was performed with a z-test. Cox proportional hazards regression stratified by transition type was used to estimate the effect of provider type on the hazard of each transition, adjusting for covariates. A significance level (alpha) of p<0.05 was used throughout all analyses. This study was approved by the Marquette University’s Institutional Review Board.

## Results

The information about 438,487 patient encounters in 325,290 subjects for NS-RCT’s was included in this study after eliminating individuals that did not meet our study criteria. In total, 105,287 subjects had a 5-year and 17,762 had a 10-year continuous follow-up, respectively. The overall survival rates for all teeth with NS-RCT were 98.19% [95% CI 98.14%, 98.23%] at 1 year, 90.83% [95% CI 90.70%, 90.95%] at 5 years, and 82.84% [95% CI 82.57%, 83.11%] at 10 years. Table 1 shows that molars received most of the NS-RCTs and anterior teeth were the least likely to receive an NS-RCT. A statistically significant difference was found in the teeth type which endodontists and the non-endodontists completed NS-RCTs (p<0.001). On average, the patients treated by endodontists were older than the patients treated by the non-endodontists.

**Table 1:** Descriptive summary of study variables

	All (n = 438487)	Endodontist (n = 138655)	Non-Endodontists (n = 299832)	p-value
**Tooth location**				<0.001^*§^
Anterior	75585 (17.2%)	14230 (10.3%)	61355 (20.5%)	
Pre-molar	121820 (27.8%)	25220 (18.2%)	96600 (32.2%)	
Molar	241082 (55.0%)	99205 (71.5%)	141877 (47.3%)	
**Age at NSRCT**				<0.001^*||^
Mean (SD)	44.7 (14.1)	46.4 (14.0)	43.9 (14.0)	
Median	46.0 (0.0, 99.0)	48.0 (0.0, 99.0)	45.0 (1.0, 96.0)	
[Min, Max]				
Freq Missing	0	0	0	
**Age at NSRCT**				<0.001^*§^
0-17	16123(3.7%)	5060 (3.6%)	11063 (3.7%)	
18-35	99319 (22.7%)	24903 (18.0%)	74416 (24.8%)	
36-53	194831 (44.4%)	61790 (44.6%)	133041 (44.4%)	
54-71	121121 (27.6%)	44159 (31.8%)	76962 (25.7%)	
71+	7093 (1.6%)	2743 (2.0%)	4350 (1.5%)	
**Gender**				< 0.001^*§^
Female	235569 (53.7%)	78833 (56.9%)	156736 (52.3%)	
Male	198065 (45.2%)	57796 (41.7%)	140269 (46.8%)	
Unknow	4853 (1.1%)	2026 (1.5%)	2827 (0.9%)	
**Core/post**				<0.001^*§^
**within 90 days**				
No core/post	161876 (36.9%)	58706 (42.3%)	103170 (34.4%)	
within 90 days				
Core/post	276611 (63.1%)	79949 (57.7%)	196662 (65.6%)	
within 90 days				
**Crown within 90 days**				<0.001^*§^
No crown within	316938 (72.3%)	101312 (73.1%)	215626 (71.9%)	
90 days				
Crown within	121549 (27.7%)	37343 (26.9%)	84206 (28.1%)	
90 days				

§ Chi-squared

* Statistically significant

Table 2 shows the number of events for each of the four possible transitions. In total, 438,487 teeth had initial NS-RCT and 407,336 (~93%) of those teeth had no additional treatments. Extraction was the most common intervention after NS-RCT followed by non-surgical retreatment. No additional treatments were found in most teeth retreated non-surgically or surgically. Extraction was a more common additional treatment than a surgical retreatment after a non-surgical retreatment.

**Table 2:** Observed number of events for each of the 4 possible transitions between the states (no failure, nonsurgical retreatment, surgical retreatment, extraction)

		Transition states		
Entering State		Nonsurgical retreatment	Surgical retreatment	Extraction
No additional treatment	407.336	4.030	1.935	25.186
Nonsurgical retreatment	4.030	–	117	422
Surgical retreatment	2.052	–	–	279
Extraction	25.887	–	–	–

**Figure 2:**
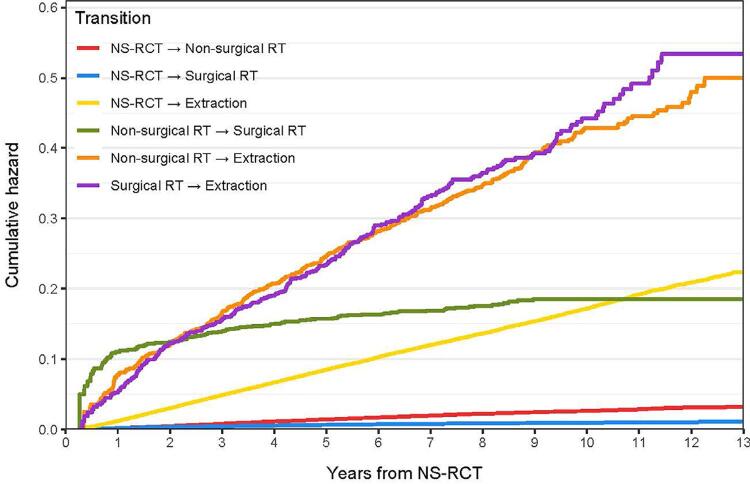
Transitions between failure states based on time from NSRCT completion

In Figure 2 (cumulative hazard plot), we report the transitions between treatment states over a 13-year study period from NS-RCT completion. In general, teeth were more likely to be extracted than retreated. The teeth which received surgical or non-surgical retreatment were more likely to be extracted than those that did not have such an intervention. Teeth were more likely to be retreated non-surgically than surgically. If a tooth had a nonsurgical retreatment and then subsequently had a surgical retreatment, then it was more likely that the surgical intervention occurred during the first year of treatment.

**Figure 3:**
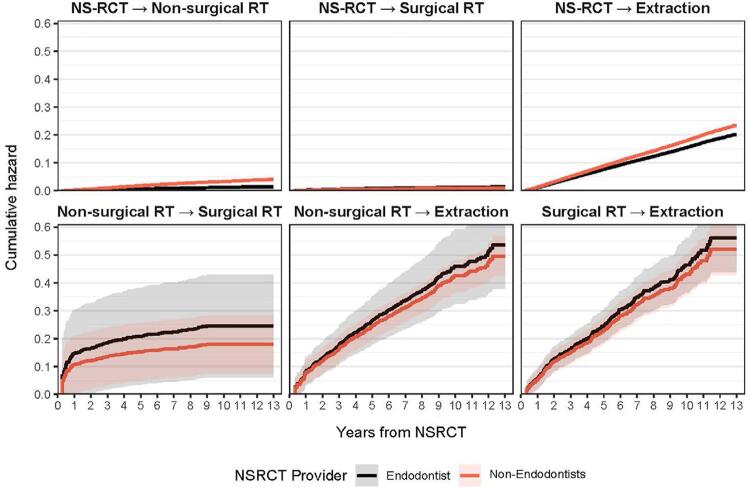
Cumulative hazard plot demonstrating time to the next transition state based on initial provider type

Figure 3 reports the effect of initial provider type on time to the next transition state (additional treatment). The probability of nonsurgical retreatment or extraction as the first additional treatment after NS-RCT was higher when the initial NS-RCT was performed by non-endodontists. The confidence intervals (depicted as shaded areas around the trend lines) for the other transitions we examined were too wide to draw definitive conclusions. Hence, the differences in transitions from nonsurgical retreatment to surgical retreatment, nonsurgical retreatment to extraction, and surgical retreatment to extraction were not significantly different based on the provider type.

In Table 3, we report the transition probabilities for teeth that received initial NS-RCT from endodontists and non-endodontists. The probability of teeth receiving further treatments after initial NS-RCT is very low irrespective of the type of provider in the 12-year follow-up period. Only a small number of teeth had subsequent interventions. In case of both types of providers, extraction was the most common type of intervention. The probability of a tooth with NS-RCT being retreated (non-surgically or surgically) or extracted was higher when the initial NS-RCT was completed by non-endodontists (Table 3).

**Table 3:** Probability (SE) of being in each state at select time-points

Timepoint	State	Endodontist	Other providers	Difference	p-value
2-year	No additional treatment	96.7% ( 0.0%)	96.1% ( 0.0%)	0.7% ( 0.1%)	<0.0001
	Nonsurgical retreatment	0.2% ( 0.0%)	0.5% ( 0.0%)	-0.3% ( 0.0%)	<0.0001
	Surgical retreatment	0.4% ( 0.0%)	0.3% ( 0.0%)	0.1% ( 0.0%)	<0.0001
	Extraction	2.7% ( 0.0%)	3.1% ( 0.0%)	-0.4% ( 0.0%)	<0.0001
4-year	No additional treatment	93.0% ( 0.1%)	91.5% ( 0.1%)	1.6% ( 0.1%)	<0.0001
	Nonsurgical retreatment	0.4% ( 0.0%)	1.2% ( 0.0%)	-0.8% ( 0.0%)	<0.0001
	Surgical retreatment	0.6% ( 0.0%)	0.5% ( 0.0%)	0.1% ( 0.0%)	<0.0001
	Extraction	5.9% ( 0.1%)	6.8% ( 0.1%)	-0.9% ( 0.1%)	<0.0001
6-year	No additional treatment	89.7% ( 0.1%)	87.4% ( 0.1%)	2.3% ( 0.1%)	<0.0001
	Nonsurgical retreatment	0.6% ( 0.0%)	1.7% ( 0.0%)	-1.1% ( 0.1%)	<0.0001
	Surgical retreatment	0.8% ( 0.0%)	0.6% ( 0.0%)	0.2% ( 0.0%)	<0.0001
	Extraction	9.0% ( 0.1%)	10.3% ( 0.1%)	-1.4% ( 0.1%)	<0.0001
8-year	No additional treatment	86.6% ( 0.1%)	83.7% ( 0.1%)	3.0% ( 0.2%)	<0.0001
	Nonsurgical retreatment	0.8% ( 0.0%)	2.1% ( 0.1%)	-1.4% ( 0.1%)	<0.0001
	Surgical retreatment	0.8% ( 0.0%)	0.7% ( 0.0%)	0.2% ( 0.1%)	0,00061
	Extraction	11.8% ( 0.1%)	13.5% ( 0.1%)	-1.8% ( 0.2%)	<0.0001
10-year	No additional treatment	83.7% ( 0.2%)	80.2% ( 0.2%)	3.5% ( 0.2%)	<0.0001
	Nonsurgical retreatment	0.8% ( 0.1%)	2.3% ( 0.1%)	-1.5% ( 0.1%)	<0.0001
	Surgical retreatment	0.9% ( 0.1%)	0.7% ( 0.0%)	0.2% ( 0.1%)	0,0043
	Extraction	14.6% ( 0.2%)	16.8% ( 0.1%)	-2.2% ( 0.2%)	<0.0001
12-year	No additional treatment	80.7% ( 0.2%)	76.7% ( 0.2%)	4.0% ( 0.3%)	<0.0001
	Nonsurgical retreatment	0.9% ( 0.1%)	2.7% ( 0.1%)	-1.7% ( 0.1%)	<0.0001
	Surgical retreatment	0.9% ( 0.1%)	0.7% ( 0.0%)	0.2% ( 0.1%)	0,019
	Extraction	17.5% ( 0.2%)	20.0% ( 0.2%)	-2.5% ( 0.3%)	<0.0001

Table 4 shows the results from the proportional hazards model to estimate the effect of provider adjusting for other covariates on transitions. The hazard of transitioning from NS-RCT to non-surgical RT and NS-RCT to extraction was significantly lower when the provider was an endodontist. However, endodontist treated teeth did have a slightly higher risk of the transition from NS-RCT to surgical RT although the hazard was low. The hazard of transition from NS-RCT to non-surgical RT decreases with increasing age, although, the hazard increases with age for the other transitions we examined. Males had lower hazard of transition from NS-RCT to either of the retreatments but a higher hazard for extraction when compared with females. In general, anterior teeth had a lower hazard to transition to non-surgical RT and a greater hazard to transition to surgical retreatment or extraction indicating posterior teeth are more likely to undergo an extraction than a surgical RT. Teeth that received a post/core or crown within 90 days after completion of initial NS-RCT had better outcomes than teeth which had the restorations after 90 days.

**Table 4:** Proportional hazards model to estimate the effect of provider adjusting for other covariates on transitions

Predictor	Comparison	NS-RCT -> Non-surgical RT	NS-RCT -> Surgical RT	NS-RCT -> Extraction	Non-surgical RT -> Surgical RT	Non-surgical RT -> Extraction	Surgical RT -> Extraction
Group	Other provider vs endodontist	3.27 ( 2.98-3.58), p<0.0001	0.64 ( 0.58-0.71), p<0.0001	1.30 ( 1.27-1.34), p<0.0001	0.58 ( 0.34-0.99), p=0.047	0.94 ( 0.70-1.27), p=0.69	1.10 ( 0.85-1.41), p=0.48
age	Age increase in 10 year increments	0.93 ( 0.91-0.95), p<0.0001	1.11 ( 1.08-1.15), p<0.0001	1.19 ( 1.18-1.20), p<0.0001	1.17 ( 1.02-1.34), p=0.025	1.25 ( 1.15-1.36), p<0.0001	1.08 ( 0.97-1.20), p=0.14
Gender	Male vs female	0.86 ( 0.81-0.92), p<0.0001	0.80 ( 0.73-0.87), p<0.0001	1.04 ( 1.02-1.07), p=0.0017	1.71 ( 1.18-2.46), p=0.0044	1.05 ( 0.86-1.27), p=0.63	0.80 ( 0.63-1.03), p=0.086
Tooth Location	Pre-molar vs anterior	1.03 ( 0.92-1.15), p=0.65	0.48 ( 0.42-0.54), p<0.0001	1.03 ( 0.99-1.08), p=0.095	0.26 (0.15-0.43), p<0.0001	0.85 ( 0.59-1.23), p=0.38	1.63 ( 1.11-2.39), p=0.012
Tooth Location	Molar vs anterior	2.06 ( 1.87-2.27), p<0.0001	0.38 ( 0.34-0.42), p<0.0001	1.25 ( 1.20-1.29), p<0.0001	0.21 ( 0.14-0.32), p<0.0001	1.06 ( 0.77-1.46), p=0.71	2.76 ( 1.97-3.86), p<0.0001
CR90	Crown within 90 days vs not	0.77 ( 0.71-0.83), p<0.0001	0.83 ( 0.74-0.92), p=0.00068	0.50 ( 0.49-0.52), p<0.0001	0.97 ( 0.58-1.62), p=0.91	1.14 ( 0.89-1.45), p=0.30	1.82 ( 1.40-2.37), p<0.0001
CP90	Core/post within 90 days vs not	0.89 ( 0.84-0.95), p=0.00044	1.08 ( 0.98-1.19), p=0.11	0.74 ( 0.72-0.76), p<0.0001	1.37 ( 0.92-2.02), p=0.12	1.05 ( 0.85-1.29), p=0.65	0.85 ( 0.66-1.09), p=0.20

## Discussion

This study aimed to determine the transitions by intermediate events along multiple paths that teeth with NS-RCT may undergo. In our study, most (~93%) teeth with NS-RCTs had no additional treatment. This is similar to the findings of Lazarski, et al.^14^ (2001), who found 94.44% of teeth with NS-RCT were functional over an average follow-up time of 3.5 years. When root canal therapy fails, the patient and provider decide if the best course of treatment is endodontic retreatment or extraction. The goal for the retreatment, besides removing the old filling, treating missed canals and improving any kind of shortcomings of the previous treatment is the same as the initial therapy, which is to remove the infection and create a favorable environment for healing. Deciding how to proceed after an endodontic failure is complex. The quality of the previous treatment and/or the restoration determines the further course of treatment after endodontic failure.^12,15^ If the provider determines that they can improve the quality of the initial root canal therapy and navigate previously unaddressed canal space without drastically weakening the tooth structure, then the treatment decision would be to retreat non-surgically.^15^

Nonsurgical retreatment is considered as the first line of treatment for an endodontic failure if the tooth is restorable.^16^ This study identified a greater likelihood of a nonsurgical retreatment than a surgical retreatment. We found that approximately 13% of secondary treatments had a subsequent intervention which would indicate a failure of the treatment. This is similar to a study by Ng, Mann, Gulabivala^17^ (2008) in a systematic review, which reported a success rate of 77% for secondary root canal treatment.^17^ The multi-state analysis also found that both non-surgically retreated or surgically retreated teeth had similar probabilities of being extracted, which was greater than teeth that did not have secondary treatment after the NS-RCT. This finding differs from the meta-analysis by Torabinejad, et al.^13^ (2009), which found that endodontic surgery offers more favorable initial success and nonsurgical retreatments have favorable long-term outcomes.^13^ On the other hand, our results agree with the meta-analysis conducted by Del Fabbro, et al.^12^ (2016), which found no significant differences in the long-term outcomes between surgical and non-surgical retreatments.^12^Haxhia, et al.^18^ (2021) also found no differences in outcomes between non-surgically retreated or surgically retreated teeth.

The most common intervention after NS-RCT was the extraction of the involved tooth, a finding similar to what was previously reported.^3,10,14^ Among the studies which examined the reasons for tooth exactions after endodontic treatment, Touré, et al.^19^ (2011) reported that the reasons for extraction were periodontal disease in 40.3% and endodontic failure in 19.3% of cases, whereas Chen, et al. ^20^ (2008) reported that only 10% of the extractions were due to endodontic failure. Extracting endodontically treated teeth may be due to non-restorability, patient finances, crown or root fractures, or provider philosophy. Clinicians may lack confidence in the success of retreatment therapy leading to increased pressure to replace “failed” endodontically treated teeth with implants.^21^ However, Kim found that, after primary endodontic failure, the most cost-effective treatment was microsurgery. This was followed by nonsurgical retreatment, then extraction and fixed partial denture, and the least cost-effective treatment was a single unit implant.^22^ Nevertheless, in our study, more teeth with failed NS-RCT were extracted than retreated.

When considering transitions to additional treatment states, the cumulative hazard for most transitions accumulates at an almost constant rate, implying that the risk of these events does not change over time. The transition from non-surgical to surgical retreatment has a very different shape: most of the cumulative hazard accumulates within a year, with relatively little increase afterwards. That is, if a surgical retreatment did not happen within a year after the non-surgical retreatment, it is unlikely to happen afterwards. Obviously, additional treatment after nonsurgical retreatment indicates failure of the procedure. The failure could be due to reasons such as improper identification of reasons for failure of NS-RCT such as presence of a vertical root fracture (VRF) or faulty retreatment procedures. In any case, we suspect the greater hazard of transition recorded in the first year is probably related to the general practice of treatment follow ups at the 3-6 month and 1-year interval for these cases leading to identification of the failure and provision of additional treatment.

In our covariate adjusted MSM, we examined the relative impact of variables such as the type of provider, age of the patient, type of tooth and time to restoration on the transitions. The type of provider influenced the transitions that a NS-RCT treated tooth underwent. At every follow-up point, a slightly higher probability of teeth treated by non-endodontists to receive additional intervention was identified. Endodontist treated teeth had a slightly higher risk of the transition to surgical RT. However, the risk is very low. In a previous report published using the same dataset, Burry, et al.^4^ (2016) found better treatment success when the provider was an endodontist. We found a decrease in the risk of transition from NS-RCT to non-surgical RT with an increase in age indicating a lower probability of failure among older individuals. A meta-analysis by Kojima, et al.^23^ (2004) found no significant difference between age groups in endodontic success.^23^ However, Ørstavik, et al.^24^ (2004) had a finding similar to our study. They found that the results were better for the older age groups. They postulated that progressive reduction of pulp space with age limits space for infection and makes it easier to provide adequate canal debridement and filling.^24^ We found that the risk of retreatments after NS-RCT was lower among men although they had a marginally higher risk for extraction than women. A meta-analysis and a prospective study both by Ng, et al.^25, 27^ (2008, 2011), as well as the Toronto Study,^27^ reported no significant differences in success of NS-RCT between men and women. Our analysis also found that the presence of permanent restoration within 90 days after the NS-RCT had influenced the treatment transitions of the tooth with generally positive outcomes among those who had a post/core, and crown within 90 days of NS-RCT. This confirms findings from previous studies by Yee, et al.^5^ (2018), Salehrabi and Rotstein (2004) and Lazarski, et al.^14^ (2001).

To the best our knowledge, we presented the first application of a multi-state model to data from subjects with NS-RCT aiming to introduce the advantages of this type of analysis of outcomes of dental treatments. In treatment outcome studies in which survival at a certain point of time in the future is the outcome of interest, survival generally has two states and one possible transition from a survival state (alive) to a failure state (dead).^11^ However, success/failure of endodontic treatments may be subdivided into two or more transitional (intermediate) states, each corresponding to a progressively more complex/invasive treatment. The possible transition states are: no additional treatment, non-surgical retreatment, surgical retreatment, and extraction. In these cases, transition of patients through the various states can be modelled by using MSM that can allow individuals to move randomly among a finite number of states. For our study, only transitions to a higher level of re-intervention were allowed, since once a tooth receives a treatment or retreatment, the next treatment is typically more complex. For example, surgically retreated tooth will typically go for another surgical retreatment or extraction and rarely undergo a non-surgical retreatment.

We excluded patients that had a follow-up shorter than 90 days (34,616) after their initial NS-RCT. Having a definitive restoration within a reasonable timeframe after NS-RCT is a strong predictor of survival of endodontically treated teeth. Hence, we wanted to incorporate having a core/post and crown by 90-days as predictors. The easiest way to achieve this objective was to start counting from 90 days, since this would eliminate any ambiguity about the status of the restoration. We also excluded 3,376 patients with a failed NS-RCT in the first 90 days after the procedure which is 0.7% of teeth with NS-RCT. This translates to an annual incidence of 2.7% which is higher than the incidence of extractions during the remaining study period. We suspect this may be due to errors in NS-RCT technique (e.g., missed canals) or improper case selection (e.g., cracked teeth) which can lead to persistence or aggravation of symptoms, leading to an extraction.

This study has a very large study population, which allowed us to evaluate the true outcome of teeth including all the endodontic treatments that occurred during the tooth “lifespan” by performing the DDWI entire database for an extended period (a 13-year period). This large study population also allows the statistical analysis to detect minor departures from the null hypothesis. The immense dataset can minimize the effects of variations in treatment or providers. It also provides a way to study tooth survival and true outcomes of teeth treated by NS-RCT in the real world. Many studies are performed in residency programs or evaluating smaller groups of private practices. An important limitation of these studies is that they are only representative of their office and the treatment and decisions by their referring dentists.^26^ With this study, we have access to the true outcome of teeth treated across the entire state of Wisconsin with a broad variety of patients and providers. As a retrospective analysis of administrative data, it eliminates any provider-related biases in treatment planning decisions. This allows for this study to yield pragmatic outcomes and provides information as the treatment be provided to a large population.

In retrospective insurance studies, is impossible to have standardization of the providers or attempt to understand the rationale for a treatment decision. They cannot provide insight into the quality of treatment provided or if proper techniques were followed. The inability to understand the rationale for treatment can result in underestimated survival, since providers may be extracting teeth that are otherwise restorable or choosing not to retreat a tooth that may have a good chance of success in favor of an implant. It is also impossible to consider additional factors that may affect the survival such as the periodontal health of the patient, pulpal and periradicular diagnosis of the tooth or remaining tooth structure before the. Additionally, considering that this study is evaluating survival, the teeth studied that have survived may not actually be a true successful treatment. For example, in instances that the teeth could have asymptomatic lesions associated with them or in cases in which patients have not received needed treatments. Finally, DDWI is a private insurance carrier in one state and the results may not be generalizable to other populations.

## Conclusion

Most teeth remained in the same state after treatment with no additional treatment transitions. When a transition occurred, it was more likely to be an extraction. Type of provider, age, location of the tooth, gender, and time to placement of final restoration significantly influence treatment transitions.
